# Unexplored parameters of ulnar nerve in the palm and its clinical implications; A cadaveric study

**DOI:** 10.1016/j.amsu.2022.103259

**Published:** 2022-01-14

**Authors:** Rohini Punja, Gaurav Kini, Mamatha Hosapatna

**Affiliations:** a Department of Anatomy, Kasturba Medical College, Manipal, Manipal Academy of Higher Education, Manipal, Karnataka, 576104, India; b Kasturba Medical College, Manipal, Manipal Academy of Higher Education, Manipal, Karnataka, 576104, India

**Keywords:** Compression, Guyon's canal, Metacarpal head, Pisiform, Ulnar nerve

## Abstract

**Objectives:**

Several studies have been conducted on the variations and branching pattern of the ulnar nerve in the hand. There are few studies conducted on defining the distance of ulnar nerve from bony landmarks in the palm. Ulnar nerve is closely related to the pisiform and hook of hamate which act as important landmarks.

**Methods:**

The study was conducted on 30 formalin fixed adult hand specimens in the department of Anatomy. Various measurements related to the ulnar nerve in the palm were taken using a divider and Vernier Calipers and the values were tabulated after obtaining the mean and standard deviation.

**Results:**

The average distance seen in the hand specimens [n = 30] from pisiform to the division of ulnar nerve into superficial and deep branch was 0.89 ± 0.25cm and the distance between pisiform bone up to the division of superficial branch of ulnar nerve into proper and common digital branches was 1.36 ± 0.59 cm. The average distance from the origin of proper digital branch of ulnar nerve to the head of fifth metacarpal bone was 5.25 ± 0.59 cm. The length of common digital branch of ulnar nerve from its origin to division into 2 sensory branches was 4.31 ± 1.09 cm.

**Conclusion:**

This study provides the metric parameters of the ulnar nerve in the hand from its significant bony landmarks which should be kept in mind during surgical procedures to minimize the incidence of injury to its branches. It would assist the orthopedic surgeon in the treatment of ulnar nerve compression in the Guyon's canal.

## Highlights

• Ulnar nerve is closely related to the pisiform and hook of hamate.
• Occupation related injuries seen in clerical, industrial and professional cyclists.
• Distance of ulnar nerve from pisiform 0.89 ± 0.25 cm.
• Length of common digital branch was 4.31 ± 1.09 cm.
• Distance from bony landmarks minimize the incidence of injury to its branches.
• Knowledge beneficial during treatment of nerve compression in Guyon’s canal.

## 1 Introduction

The ulnar nerve [UN] is the continuation of medial cord of brachial plexus with a root value of C8, T1 and often receives fibers from C7. The UN accompanied by the ulnar artery passes within the Guyon's canal after giving off the dorsal and palmar cutaneous branches. The UN is closely related to the pisiform and hook of hamate which act as important landmarks [1].

Distal to the pisiform but proximal to the hook of the hamate UN divides into superficial sensory and deep motor branches. The digital nerves given off by the superficial branch of UN innervate the fourth and fifth digits. These digital branches are given off distal to the hook of hamate [1,2]. The deep branch supplies most of the intrinsic muscles of the hand and terminates by supplying adductor pollicis [3]. Variations of the UN in the hand have been reported in numerous articles by various authors [4].

Occupation related injuries and disorders related to the UN can be seen in clerical, industrial and professional cyclists [5,6]. The palsy affecting long-distance cyclist involving the UN in the Guyon's canal is sometimes referred to as “cyclist's/handlebar palsy” [6]. The incidence of neuropathy involving the UN is around 20.9%. Entrapment of the UN in the cubital tunnel is common compared to that at the Guyon's canal [7].

It has been stated that knowledge of UN variations in the volar aspect of the hand are important and can justify the reason for pain and sensory loss in patients’ after surgical procedures and injuries [8]. We have conducted this study to emphasis more on the morphometric parameters of the UN in relation to the important bony landmarks such as the pisiform and head of metacarpal to aid the hand surgeons during various procedures such as entrapment syndromes and neuropathies.

## 2 Materials and methods

**Fig. 1:**
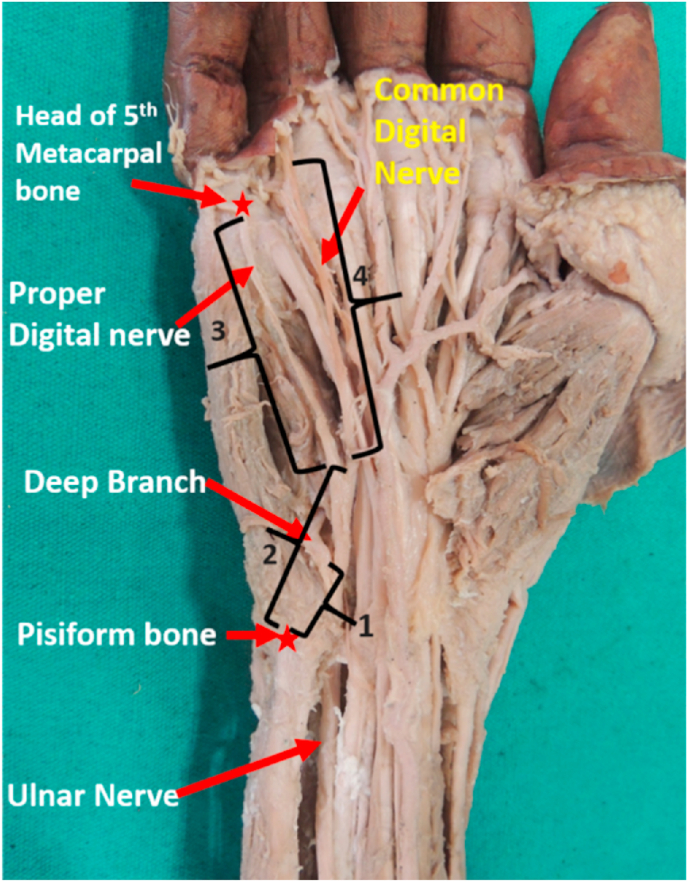
Right hand specimen illustrating the various morphometric parameters of the UN measured from the bony landmarks. [1- Distance between pisiform bone and division of ulnar nerve into terminal branches; 2- Distance between pisiform bone and division of superficial branch of ulnar nerve; 3- Distance between head of fifth metacarpal bone and proper digital branch of ulnar nerve; 4- Length of common digital branch of ulnar nerve from its formation to division into 2 sensory branches].

This cross sectional cadaveric study was conducted on 30 formalin fixed dissected hand specimens obtained from Department of Anatomy. The study was conducted under the Code of Ethics in accordance with the Declaration of Helsinki for experiments involving humans. The following lengths were measured in the palm using a divider and Vernier Calipers [Fig. 1]:

1) Distance between pisiform bone and division of UN into terminal branches
2) Distance between pisiform bone up to the division of superficial branch of UN into proper and common digital branches.
3) Distance from the origin of proper digital branch of UN to the head of fifth metacarpal bone.
4) Length of common digital branch of UN from its origin to division into 2 sensory branches.

## 3 Results

The average distance seen in all the hand specimens [n = 30] from pisiform to the division of UN into superficial and deep branch was 0.89 ± 0.25 cm and the distance between pisiform bone up to the division of superficial branch of UN into proper and common digital branches was 1.36 ± 0.59 cm. The average distance from the origin of proper digital branch of UN to the head of fifth metacarpal bone was 5.25 ± 0.59 cm. The length of common digital branch of UN from its origin to division into 2 sensory branches was 4.31 ± 1.09 cm. The above measurements for the right [n = 16] and left [n = 14] side individually have been mentioned in Table 1.

**Table 1:** The various measurements of ulnar nerve in palm for the right and left side.

Measured Parameters	Right[n = 16]	Left[n = 14]
Distance between pisiform bone and division of ulnar nerve.	0.96 ± 0.29	0.80 ± 0.16
Distance between pisiform bone up to the division of superficial branch of ulnar nerve into proper and common digital branches	1.49 ± 0.64	1.208 ± 0.521
Distance from the origin of proper digital branch of ulnar nerve to the head of fifth metacarpal bone	5.09 ± 0.691	5.44 ± 0.37
Length of common digital branch of ulnar nerve from its origin to division into 2 sensory branches	4.06 ± 1.26	4.60 ± 0.81

## 4 Discussion

Guyon's canal acts as a potential site for compression neuropathies of the UN in the hand which could be due to occupation related trauma or fractures involving the carpal bones-pisiform/hook of hamate [9]. Intraoperative involvement of the UN during treatment of ulnar artery thrombosis or ganglion cyst/tumor could be another complication [10].

Sulaiman S et al. in their cadaveric study measured distance between pisiform bone and division of UN [13.6 ± 4.0 mm] and distance between pisiform bone up to the division of superficial branch of UN into proper and common digital branches [25.2 ± 4.6 mm]. He stated that these indices of locating the UN and its branches using a bony, palpable landmark, would aid surgeons working in this region to avoid injury as well as plan new surgical approaches [11]. Verhiel et al. measured the median distance between the wrist flexion crease to the deep branch of UN which was 12 mm [12]. The median distance between the wrist flexion crease to the division of superficial branch of UN into proper and common digital branches was 24 mm [12]. Whereas in the present study we measured the same distance but considered a bony landmark, the pisiform bone, instead of the wrist flexion crease since it is prominent and easy to identify. The measurements were 0.89 ± 0.249 and 1.36 ± 0.595 respectively. Pisiform's proximity to the ulnar nerve in the Guyon canal, acts as a factor which could compress the nerve in case of fracture to this bone [13].

These morphometric parameters maybe useful for hand surgeons during surgical procedures such as carpal tunnel release, palmar fasciectomy, and tendon release on the flexor aspect of hand [11,14,15] in identifying and retaining the important branches present on the palmar aspect of the hand. In an attempt to provide topographical information about these vital structures in the hand several authors have considered various landmarks as a reference point such as the metacarpophalangeal joint, bistyloid line, superficial palmar arch [11,14,[16], [17], [18]].

The superficial branches of UN namely the proper and common digital branch could be used as nerve grafts since literature shows the use of dorsal branch of ulnar nerve [19] hence in the present study we have measured the distance from the origin of proper digital branch of UN to the head of fifth metacarpal bone and length of common digital branch of UN from its origin to division into 2 sensory branches.

**Fig. 2:**
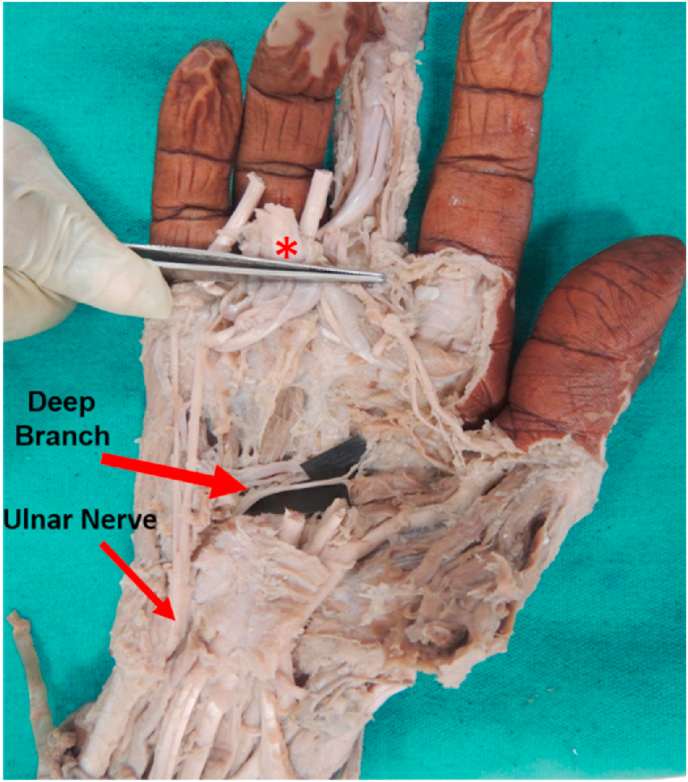
Right hand specimen illustrating the deep branch of ulnar nerve terminating by supplying adductor pollicis [*cut end of tendons of superficial and deep flexor muscles reflected up].

Our study follows the classical pattern of branching of the UN which states that it divides into the superficial and deep branches as reported by many authors [2,16,20]. The deep branch which is a motor branch supplies most of the intrinsic muscles of the hand and ends by supplying the adductor pollicis. The same was observed in our study for all the 30 hand specimens as shown in Fig. 2.

Since UN in the hand is a mixed nerve compression close to the pisiform bone may involve both the motor as well as sensory distribution thus the precise knowledge of the division of UN from the pisiform plays an important role. De-roofing the Guyon's canal in surgical release of the UN at the wrist may damage the neurovascular structures [21]. Wrist surgeries these days are performed arthroscopically and the knowledge as to where to put the portals and the important anatomic structure nearby is essential for these surgeons. Thus, knowledge of these structures from its bony landmark may prevent accidental entry into Guyon's canal [21].

Certain limitations of our study are that this study was done on formalin fixed specimens and findings could not be compared with the other respective hand [same cadaver] specimens. Another limitation of this study is that we did not observe the communicating branch between the digital branches of UN and median nerve which have been done by several authors [22].

## 5 Conclusion

This study provides the metric parameters of the ulnar nerve in the hand from its significant bony landmarks which should be kept in mind during surgical procedures to minimize the incidence of injury to its branches. Our study explored some of the landmarks which have not been considered previously and could aid in identifying the UN and its branches in the palm. We have used pisiform bone and head of fifth metacarpal as bony landmarks for measuring the length of various branches of UN in the palm. Previously no such study has been done and this knowledge would be beneficial to hand surgeons.

## Provenance and peer review

Not commissioned, externally peer reviewed.

## Ethical approval

Since the study involved dissected limbs used for MBBS students ethical committee clearance was not obtained during the study

## Please state any sources of funding for your research

As this study was on conducted on cadavers no funding sources for this project.

## Author contribution

GK was involved in conception and design of the work; acquisition, analysis, interpretation of data for the work and wrote the initial and final draft of article. MH and SKS conceived and designed the study, conducted research, provided research materials, collected, and organized data, wrote the initial draft of article. RP was involved in drafting the work, revising it critically for important intellectual content and the final approval of the version. All authors have critically reviewed and approved the final draft of the manuscript.

## Registration of research studies

Name of the registry: not applicable

Unique Identifying number or registration ID:

Hyperlink to your specific registration (must be publicly accessible and will be checked):

## Guarantor

Dr.Mamatha H.

Additional professor.

Department of Anatomy.

KMC Manipal.

## Consent

Since the study involved dissected limbs used for MBBS students ethical committee clearance was not obtained during the study.

## Declaration of competing interest

No conflicts of interest.

## References

[1] Gray H., Standring S., Ellis H., B Berkovitz B.K. (2005).

[2] Gross M.S., Gelberman R.H. (1985). The anatomy of the distal ulnar tunnel. Clin. Orthop. Relat. Res..

[3] Reckelhoff K.E., Jinpu L., Martha A.K., Daniel W.H., Norman W.K. (2015). Ultrasound evaluation of the normal ulnar nerve in Guyon's tunnel: cross-sectional area and anthropometric measurements. J. Med. Ultrasound.

[4] Gindha G.S., Kaushal S., Kalyan G.S., Sharma A. (2013). Variation in termination of ulnar nerve in hand(trifurcation of ulnar nerve). J. Adv. Res. Biol. Sci..

[5] Marcus M., Gerr F., Monteilh C. (2002). A prospective study of computer users: II. Postural risk factors for musculoskeletal symptoms and disorders. Am. J. Ind. Med..

[6] Patterson J.M., Jaggars M.M., Boyer M.I. (2003). Ulnar and median nerve palsy in long-distance cyclists, A prospective study. Am. J. Sports Med..

[7] Mondelli M., Giannini F., Ballerini M., Ginanneschi F., Martorelli E. (2005). Incidence of ulnar neuropathy at the elbow in the province of Siena (Italy). J. Neurol. Sci..

[8] Paraskevas G., Gekas C., Tzaveas A., Spyridakis I., Stoltidou A., Ph Tsitsopoulos P. (2008). Kaplan anastomosis of the ulnar nerve: a case report. J. Med. Case Rep..

[9] Aguiar P.H., Bor-Seng-Shu E., Gomes-Pinto F. (2001). Surgical management of Guyon's canal syndrome, an ulnar nerve entrapment at the wrist: report of two cases. Arq. Neuropsiquiatr..

[10] Haferkamp H. (1998). Ulnariskompression im Bereich der Handwurzel (Ulnar nerve compression in the area of the wrist). Langenbecks Arch. Chir. Suppl. Kongressbd..

[11] Sulaiman S., Soames R., Lamb C. (2015). Ulnar nerve cutaneous distribution in the palm: application to surgery of the hand. Clin. Anat..

[12] L Verhiel S.H.W., Hooven D.V., Garg R., Gottlieb R.E.W., F Ritt M.J.P., Chen N.C., Eberlin K.R. (2019). Patterns of ulnar nerve arborization in the palm: clinical implications for nerve decompression in the hand and wrist. J. Hand Surg. Glob. Online.

[13] Chang M.K., Yap R.T.J. (2019). Acute ulnar nerve compression associated with pisiform fracture - a case report and literature review. Case Rep. Plast. Surg. Hand Surg..

[14] Stancić M.F., Mićović V., Potocnjak M. (1999). The anatomy of the Berrettini branch: implications for carpal tunnel release. J. Neurosurg..

[15] Boughton O., Adds P.J., Jayasinghe J.A. (2010). The potential complications of open carpal tunnel release surgery to the ulnar neurovascular bundle and its branches: a cadaveric study. Clin. Anat..

[16] Bonnel F., M Vila R. (1985). Anatomical study of the ulnar nerve in the hand. J. Hand Surg. Br..

[17] Loukas M., Jr Louis R.G., Stewart L. (2007). The surgical anatomy of ulnar and median nerve communications in the palmar surface of the hand. J. Neurosurg..

[18] Ferrari G.P., Gilbert A. (1991). The superficial anastomosis on the palm of the hand between the ulnar and median nerves. J. Hand Surg. Br..

[19] Usami S., Kawahara S., Inami K., Hirase Y. (2019). Use of a vascularized dorsal sensory branch of an ulnar nerve flap for repairing a proper digital nerve with coverage of a volar soft tissue defect: report of two cases. Microsurgery.

[20] Murata K., Tamai M., Gupta A. (2004). Anatomic study of variations of hypothenar muscles and arborization patterns of the ulnar nerve in the hand. J. Hand Surg. Am..

[21] Blum A.G., Zabel J.P., Kohlmann R. (2006). Pathologic conditions of the hypothenar eminence: evaluation with multidetector CT and MR imaging. Radiographics.

[22] Biafora S.J., Gonzalez M.H. (2007). Sensory communication of the median and ulnar nerves in the palm. J. Surg. Orthop. Adv..

